# Effectiveness of a patient-specific 3-dimensional printed model in Septal Myectomy of hypertrophic cardiomyopathy

**DOI:** 10.12669/pjms.36.7.2620

**Published:** 2020

**Authors:** Yang Wang, Hongchang Guo, Shengwei Wang, Yongqiang Lai

**Affiliations:** 1 Yang Wang, Department of Cardiac Surgery, Beijing Anzhen Hospital, Capital Medical University, Beijing, P.R. China; 2 Hongchang Guo, Department of Cardiac Surgery, Beijing Anzhen Hospital, Capital Medical University, Beijing, P.R. China; 3 Shengwei Wang, Department of Cardiac Surgery, Beijing Anzhen Hospital, Capital Medical University, Beijing, P.R. China; 4 Yongqiang Lai, Department of Cardiac Surgery, Beijing Anzhen Hospital, Capital Medical University, Beijing, P.R. China

**Keywords:** Heart model, Hypertrophic cardiomyopathy, 3D printing, Myectomy

## Abstract

**Objective:**

Advanced cardiovascular surgery in structural heart disease require accurate pre-operative evaluation. Most of non-invasive imaging technologies remain limited in two-dimensional and show insufficiency of visualization for procedural planning. The aim of this study was to discuss the value of patient-specific 3-dimensional (3D) printing in treatment of hypertrophic cardiomyopathy (HCM).

**Methods:**

Patient-specific 3D-printed models were constructed preoperatively in 12 consecutive HOCM patients which come to Beijing Anzhen Hospital for surgical treatment from October 2016 to March 2017. Image files were extracted from multi-slice computed tomography images, 3D models were constructed by the Mimics 19.0 software and generated by Objet350 Connex3 3D printer. The 3D-printed models were made with soft material that can be surgically performed. The modified Morrow myectomy of the model was performed before the operation. Clinical characters and echocardiographic parameters were recorded.

**Results:**

There was no significant difference in tissue volume between the models and specimens. Preoperative and postoperative echocardiography showed the septal thickness was reduced from 18.8±4.5 mm to 12.7±3.3 mm (p<0.001), the left ventricular outflow tract obstruction was adequately relieved (83.0±27.73 mm Hg to 8.7±6.5 mm Hg, p<0.001), and the SAM disappeared completely after the operation. Cardiac function was improved in all patients (New York Heart Association functional class III to class I/II).

**Conclusions:**

The proposed optimal 3D-modelled septal myectomy allows intraoperative monitoring of the shape and volume of the myocardium resection to achieve the ‘ideal’ interventricular septum. It eliminates obstruction in the LVOT and SAM, resulting in LV remodeling with an increase in LV end-diastolic volume and diameter at early follow-up.

## INTRODUCTION

Hypertrophic cardiomyopathy (HCM) is an inheritable cardiovascular disease characterized by unexplained left ventricular hypertrophy, left ventricular outflow tract (LVOT) obstruction, and systolic anterior motion (SAM) of the mitral valve, with a prevalence of approximately 1:500 in the general population.^1^ Patients with HCM are associated with a variety of symptom that range from dyspnea to syncope. Interventricular septum hypertrophy may focus on basal, midventricular, apical, and mixed types.^2^ The representative pathophysiology of HCM is SAM of mitral valve which contribute to the obstruction of LVOT and different degree of mitral valve regurgitation.^3^ Medical treatment is the first-line therapy for symptomatic patients with LVOT obstruction; However, septal myectomy is the procedure of choice if medical treatment is unsuccessful or intolerable.^4^,^5^ Sometimes, because the anatomy of HCM is complex, septal myectomy remains difficult to learn for young cardiac surgeons. A main challenge of septal myectomy is how to alleviate LVOT gradient completely.

Three-dimensional (3D) printing is an emerging technology that has been widely used in the medical study. Previous studies had reported that, in cardiovascular fields, 3D-printed has been used for congenital heart disease.^6^,^7^ In the present study, 3D printing models were reconstructed in 12 patients who diagnosed with hypertrophic obstructive cardiomyopathy (HOCM). We will perform septal myectomy in both 3D printing models and patients, in order to better understand the strategy of operation.

## METHODS

Twelve consecutive HOCM patients who came to Beijing Anzhen Hospital for surgical treatment from October 2016 to March 2017 were included in the study. All patients were diagnosed by echocardiography and CT scan. They were eligible for surgical procedure and met the following two criteria: (1) a LVOT gradient of ≥50 mmHg at rest or with physiological provocation; (2) unresponsive to maximum pharmacological therapy, including dyspnoea with New York Heart Association (NYHA) functional class II to IV. Patients who met the following criteria were not recommended to undergo operation: (1) aged more than 70 years (2) the presence of an additional risk for on-pump cardiac surgery, which may lead to early postoperative death. The study was approved by The Ethics Committee of Anzhen Hospital (No.AZ00305), and all patients signed informed consent.

### 3D Printing

**Fig.1:**
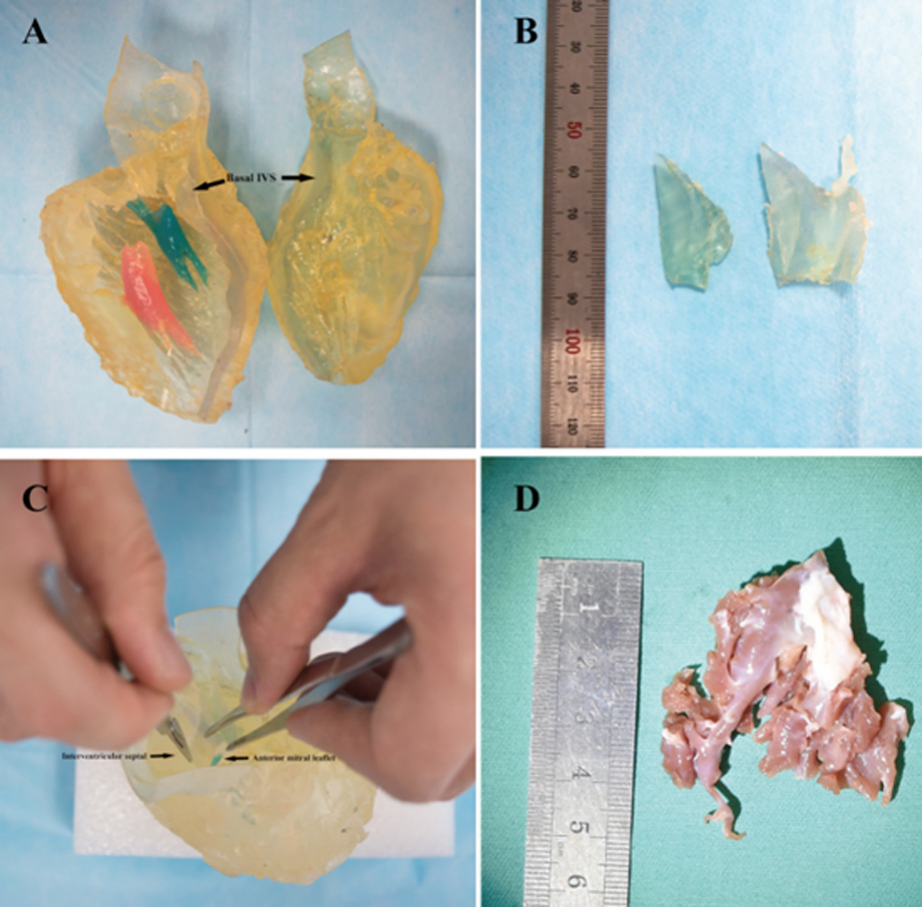
Photograph in the operating room of the 3-dimensional print and its myectomy specimens next to the actual myectomy specimen from same patient for comparison from the first patient. A) Photograph of a 3-dimensional print before operative rehearsal. B) The 3-dimensional specimens of 3-dimensional print. C) Photograph of the 3-dimensional print during operative rehearsal. D) The actual myectomy specimen from same patient.

Each patient was scanned by 64-slice spiral computed tomography (CT) scanner (GE Medical Systems, Chicago, Ill) in the supine position. The scan settings were as follows: a voltage of 120 kV, a current of 180–220 mA, and a slice thickness of 1 mm. The CT scan data of the heart were saved as DICOM files. Multilayer images were input into Mimics 19.0 (Materialize, Leuven, Belgium) to perform region reconstruction for 3D image segmentation. The models were reconstructed by the printer. After cleaning and drying of the prototype, a 1:1 ratio left heart prototype was obtained (Fig.1). Detailed information was described in our previous study.^8^

### Operative Rehearsal and Surgical techniques

**Fig.2:**
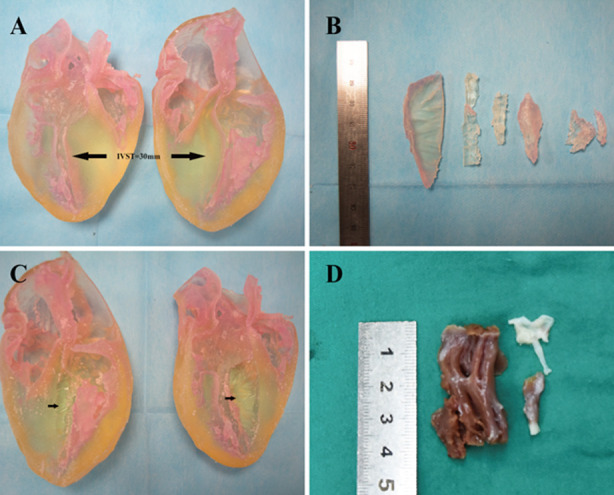
Photograph in the operating room of the 3-dimensional print and its myectomy specimens next to the actual myectomy specimen from same patient for comparison from the second patient. A) Photograph of a 3-dimensional print before operative rehearsal. B) The 3-dimensional specimens of 3-dimensional print. C) Photograph of the 3-dimensional print after operative rehearsal. D) The actual myectomy specimen from same patient.

The day before operation, the operating surgeon (Lai) performed myectomy on the 3D model. This was done in a laboratory operating room furnished with operating table and surgical lighting. Septal Myectomy of the 3D model was performed through the aorta. The 3-dimensional print before and after operative rehearsal, and its myectomy specimens from the actual myectomy specimen from same patient are shown in Fig.1,2.

After a median incision with sternotomy, cardiopulmonary bypass was performed using ascending aortic cannulation and venae cava cannulation. Septal myectomy on patients was performed through a transverse aortotomy extended into the noncoronary sinus after cardiac asystole induced with cold crystalloid cardioplegia. The left-right sinus and right nonsinus commissures were elevated and retracted with 5-0 Prolene suture. Auxiliary light and loupe magnification were used to achieve better inspection of the left ventricular cavity. The resection ranges were as follows: The upper end was located below the aortic ring; the lower end was extended to the apex of the left ventricle; from the right side, the myectomy was started slightly rightward to the nadir of the right aortic cusp; and to the left, resection was terminated near the mitral anterior commissure.^9^

### Resection Volume Measurement

A 25 mL glass graduated flask was filled with 15 mL of water and placed on the table. The part of resection from the 3D model were put into the flask and the fluctuation in level was recorded. The same graduated flask was used similarly to measure the volume of cardiac muscle resected from the patient the next day. Following operation, the 3D model and patient specimens were placed together and photographed for comparison. Since the resected 3D model and muscle specimens both sank to the bottom of the flask, the elevation in milliliters of the water level is nearly equal to the volume of the specimens regardless of any differences in density.^10^

### LVOT Gradient Measurement

Echocardiography was rountinely performed before and after operation. The view below the xiphoid were used to measure LVOT velocity by continuous-wave Doppler. Peak gradients of LVOT velocity were computed by optimal spectral Doppler envelopes and velocities computed according to the formula 4V.^2^

### Follow-up

Clinical status was obtained through phone interview with patients or family members at least yearly six months after septal myectomy. Echocardiographic data were collected during the latest clinic visits.

## RESULTS

The baseline clinical variables of HOCM patients are shown in Table-I. Seven patients reported New York Heart Association (NYHA) class III/IV heart failure symptoms. Syncope history was present in two patients. The mean septal thickness and LVOT gradient derived from TEE were (18.8±4.5) mm and (83.0±27.7) mmHg, respectively. The SAM and mitral valve insufficiency were present in all patients. (Table-I).

**Table-I:** Baseline characteristics.

Variables	Myectomy (n=12)
Age, years	41.0±13.7
BMI, kg/m^2^	24.7±3.9
Male, %	6 (50.0%)
Diabetes mellitus, %	1 (8.3%)
NYHA class III or IV, %	7 (91.7%)
Chest pain, %	3 (25.0%)
Syncope, %	2 (16.7%)
*Echocardiography*	
LVOT gradient, mmHg	83.0±27.7
IVST, mm	18.8±4.5
LVEF, %	74.8±4.9
SAM, %	12 (100%)
*CT data*	
IVST, mm	21.3±5.9

Values expressed as mean±SD, median and interquartile range, or number of patients and percentage. BMI=body mass index; LVOT, left ventricular outflow tract; IVST=interventricular septal thickness; LVEF=left ventricular fraction.

3D print prototypes were used for left heart reshaping pre- and post- operation for all patients. Represent (Fig. 1, 2) The left ventricular and the aorta were divided into two parts along left heart long axis for better vision of hypertrophic IVS, and narrowed LVOT. The papillary muscles were colored red and green. The SAM of mitral valve was obvious in the model which significantly aggravated obstruction of LVOT.

Septal myectomy was performed in all patients. The mean weight of resected muscle sample was (7.9±3.8) g and the mean cross-clamp time is (80.2±32.2) min. Septal thickness and peak gradient were (12.7±3.3) mm and (8.7±6.5) mmHg, respectively. SAM disappeared in all patients, and mitral valve regurgitation was no or trivial in five cases and mild in one patient postoperatively. The volume of resected muscle and models were similar (3.9±0.6 vs 4.1±0.3, p=0.32) (Table-II)

**Table-II:** Perioperative data.

Variables	Myectomy (n=12)
*Concomitant operation*	
Mitral valve plasty, %	4 (33.3%)
Tricuspid valvuloplasty, %	2 (16.7%)
Maze procedure, %	1 (8.4%)
Cardiopulmonary bypass time, min	130.3±57.9
Aortic clamp time, min	80.2±32.2
ICU stay, hour	70.0±37.5
Postoperative ventilation time, hour	17.0±2.5
Postoperative hospital stays, day	8.6±5.1
Pacemaker implantation, %	1(8.4%)
Weight of resected muscle, g	7.9±3.8
Mortality post-procedure (<30 days), %	0 (0%)
*Postoperative echocardiography*	
IVST, mm	12.7±3.3
LVOT gradient, mm Hg	8.7±6.5
LVEF, %	63.5±4.3
SAM, %	0 (0%)
Volume of resected muscle, ml	3.9±0.6
Volume of resected model, ml	4.1±0.3

Values expressed as mean±SD, median and interquartile range, or number of patients and percentage. BMI=body mass index; LVOT, left ventricular outflow tract; IVST=interventricular septal thickness; LVEF=left ventricular fraction.

## DISCUSSION

Completeness of septal myectomy is of great importance and the key to surgery. Many imaging techniques, including echocardiography, computerized tomography (CT) and magnetic resonance imaging (MRI), can construct two-dimension structures of the heart. Recently, an emerging technology that allows better visualization of the spatial anatomical of organs in our body from 2-dimension to 3-dimension. This new technology makes cardiothoracic surgery step further, meanwhile providing cardiac surgeon a chance for patient-specific deliberate practice of septal myectomy. This technology advances allow surgeons to evaluate surgery more accurate, however, they are just a virtual 3 dimension. Operative rehearsals with patient-specific 3D models may instruct the young surgeon a preferred septal myectomy approach, and to avoid potential pitfalls.

Previous studies had revealed that 3D printing technology can be used in congenital heart disease and aortic dissection. Shafkat et al., introduce four examples of complex congenital heart diseases using 3D technology.^11^ Kanwal et al. reported the application of 3D print to guide left ventricular assist device placement in adults with congenital heart disease and end stage heart disease.^12^ In addition, recently, Jacobs et al., reported 3D models were also used to design the resection of ventricular aneurysm and heart tumor.^13^ A few case reports had been reported, including Yang et al. reported one case of 3D-printed HOCM heart model used in septal myectomy from an apical incision.^14^

We have reconstructed the 3D models of heart of HOCM patients.^8^ The 3D models were built on a 1:1 scale, which allowed us to compare the volumes of models and patient resection specimen. In present study, we studied the relationship of operative rehearsal on 3D models and septal myectomy in 12 HOCM patients. According to the above methods, we computed the volume of the resection part from 3D models and cardiac muscles from patients. We found that the volume resected from 3D models was similar to that from the patients. The use of liquid displacement allowed the accurate measurement of materials with different densities because both were of them are nonbuoyant. The essential details that might have impact on the early results of septal myectomies are well demonstrated and better understanding preoperatively. In our study, the 3D models were constructed on a 1:1 scale in 12 patients, and allowed surgeons practice operative rehearsal from aorta view. Furthermore, the 3D models could be observed from all views, which might be very helpful for surgeon to design individual surgical, according to different types of hypertrophic interventricular septum and subvalvular abnormalities. If the volume measured from an ‘‘optimal’’ resection done on the 3D models can guide a more complete myectomy, this 3D models could help to foster improved surgical results.

Mitral valvular and sub-valvular abnormalities are popular in this patient cohort and often aggravate LVOT obstruction,^15^ and 3D models can reduce difficulty in defining and understanding the anatomy of them better preoperatively. Structural abnormalities of mitral valve, such as enlarged leaflet, leaflet elongation, or anomalous papillary muscle insertion into the anterior mitral leaflets, occurred about 66% of HOCM patients.^3^ If these anomalies can’t be recognize and managed during the operation, the residual pressure gradient after the myectomy often still exist.^16^ 3D models could illustrate these structure abnormalities individually, which could guide the surgeon to treat the associated malformations. Young surgeon who have less experience might make mistakes during this operation, including inadequate resection, and inappropriate strategy about mitral valve. If there are no structure abnormities of mitral valve, mitral valve surgery in addition to septal myectomy is not necessary as long as the myectomy performed complete.

Although 3D models can print the structure of the heart of HCM patients, it can’t simulate the changes of haemodynamics. In future studies, we will try to construct the hydrodynamic model and combine the two models together to better guide the myectomy.

### Limitations of the Study

This preliminary study is limited to only 12 HOCM patients. The 3D model in our study was reconstructed by the data derived from CT scan. The fine structures, such as chordal and mitral valve can’t simulate correctly because of lower resolution. In addition, the relationship of 3D models anatomy and hemodynamic results recorded by echocardiography will be investigated in our further study.

## CONCLUSION

Our investigation illustrates 3D-printed models derived from CT scans are feasible in patients with HOCM. The proposed optimal 3D-modelled septal myectomy allows intraoperative monitoring of the shape and volume of the myocardium resection to achieve the ‘ideal’ interventricular septum. It eliminates obstruction in the LVOT and SAM, resulting in LV remodeling with an increase in LV end-diastolic volume and diameter at early follow-up.

### Authors’ Contributions:

**YQ Lai;** Conception and design. **YQ Lai;** Administrative support. **YQ Lai**, **Y Wang**; Provision of study materials or patients. **HC Guo**, **KM Liu:** Collection and assembly of data. **YQ & YW:** Are responsible and accountable for the accuracy or integrity of the work.
